# Beatson International Cancer Conference: invasion and metastasis

**DOI:** 10.1054/bjoc.2000.1190

**Published:** 2000-04-03

**Authors:** G Stapleton, H J Spence

**Affiliations:** Beatson Institute for Cancer Research, CRC Beatson Laboratories, Garscube Estate, Switchback Road, Glasgow, G61 1BD, UK


*British Journal of Cancer* (2000) **82**(9), 1615–1617
© 2000 Cancer Research Campaign
DOI: 10.1054/ bjoc.2000.1190, available online at http://www.idealibrary.com on IDEAL®

Conference Report

### Beatson International Cancer Conference: invasion and metastasis

**G Stapleton and HJ Spence**

Beatson Institute for Cancer Research, CRC Beatson Laboratories, Garscube Estate, Switchback Road, Glasgow G61 1BD, UK

While the salient feature of cancer is uncontrolled cell growth and proliferation, the most life-threatening form of the disease occurs in the latter stages as tumours acquire the ability to invade and metastasize to other sites in the organism. During the progression to malignancy, changes in the regulation of motility and invasive properties of tumour cells occur, involving changes in response to growth factors and the extracellular matrix (ECM). Once a secondary tumour is established, its new growth and survival is dependent on the formation of new blood vessels to the tumour (angiogenesis). Importantly, all of these processes occur normally in the organism and so it is their deregulation which results in the malignant phenotype. The fourth Beatson International Cancer Conference, sponsored by the Cancer Research Campaign and the Association for International Cancer Research, provided a stimulating forum in which to emphasize the advances in cellular and molecular mechanisms controlling the various stages of metastasis, as well as highlighting the advances in potential therapeutic strategies to control these latter stages of cancer.

### CELL MOTILITY AND ACTIN REORGANIZATION

The locomotory machinery of the cell is central to both invasion and metastasis. The Rho family of GTPases connect membrane receptor signalling to cytoskeletal reorganization and the induction of motile structures. Zigmond (Philadelphia) described the role of Cdc42 and Rac in the regulation of actin polymerization dynamics, by activating the Arp2/3 complex and the nucleation of actin through Wasp. Symons (New York) proposed that while a role in transformation for Rac, Rho and Cdc42 is established, each will contribute individually to different aspects of transformation. Searches for new Rac interacting proteins revealed the family of synaptojanins whose possible functions involve endocytosis and organization of the actin cytoskeleton, however a role in Rac-dependent transformation is yet to be established. Deregulation of cell–cell interactions through the cadherin receptors is another feature of metastatic epithelial cancers, the formation and disassembly of which are controlled in part through Rho GTPases. Braga (London) highlighted a much overlooked regulatory level of signal transduction and cadherin function: the kinetics of a given signal. What is well-established regarding sustained versus transient activation of the MAP kinase pathway appears to be conserved in other signalling pathways. Whereas sustained activation of Rac in keratinocytes leads to the formation of cadherin-mediated cell–cell contacts, transient activation is required for lamellipodia formation and cell motility. Using Rac-Rho chimaeras, Braga went on to map different domains of Rac, which, presumably through the binding of different effectors, are required for cell–cell adhesion or lamellipodia formation. Cadherins signal through catenins which participate in independent signalling events through the Wnt signalling pathway. β-catenin interacts not only at the cell membrane with cadherins, but also in a complex with the LEF/TCF family of transcription factors to modulate gene expression. Birchmeier (Berlin) and Polakis (Richmond, CA) have defined the function of β-catenin in development and tumour progression and show that the regulation of β-catenin is determined by targeted degradation via ubiquination and the proteosome. Mutations in both β-catenin and the APC (adenomatous polyposis coli) gene, which is also involved in Wnt signalling and is mutated in 80% of colon cancer, affect the degradation of β-catenin and allow its accumulation in the cytosol.

The influence of the microenvironment on cell fate, function and response is of paramount importance in many cellular paradigms but certainly in aspects of cell invasion and was a recurring theme at this meeting. In particular, Gurdon (Cambridge) focused on the formation of morphogen gradients during early development where a signal generated several cell diameters away from other cells will be interpreted differently according to the concentration that a cell encounters. Cells measure their response to a given concentration by the absolute number of morphogen-bound receptors which in turn determines gene expression for low response genes versus high response genes. Studies using mammary gland further emphasized the importance of the ECM in determining normal cellular differentiation or, when deregulated, the induction of apoptosis, inappropriate morphogenesis or tumour formation (Bissell, Berkeley, CA). Gallagher (Manchester) elaborated on this theme by providing a molecular mechanism where heparin sulphate proteoglycans (HSPGs) function as sensors of the microenvironment and regulate the activation of growth factors. The degree and manner of sulphation of HSPGs determines binding and activation of FGF. 2-*O*-sulphation of the HSPG is required for binding to FGF, then further 6-*O*-sulphation promotes FGF mitogenic activity. The degree of sulphation is known to change in malignant cells, suggesting a mechanism whereby deregulated growth factor and chemokine signalling might occur.

The regulation of cell–cell and cell–ECM adhesions is a critical determinant of cell motility and Frame (Glasgow) continued on this theme, exploring the role of the proto-oncogene, c-*src*, in both types of cell adhesions. Src is localized at focal adhesions, the sites where its role appears to be in the turnover of these structures.

*Received 6 December 1999*
*Accepted 19 January 2000*

*Correspondence to:* G Stapleton

Inhibition of Src activity results in 'superstuck' cells and enlarged focal adhesions due to a lack of focal adhesion turnover. In epithelial cells, Src participates in the regulation of cell–cell contacts, where inhibition of Src activity results in their stabilization. Thus Src functions at multiple levels to regulate cell motility and invasion at both cell–cell and cell–ECM contacts. Saxton and Pawson (Toronto) addressed the regulation of receptor tyrosine kinase and integrin signalling by protein tyrosine phosphatases. Homozygous null Shp2 mice were generated which have notochord and posterior truncated phenotypes similar to the FGF8 knockout. In addition to defects in MAP kinase signalling, Shp2 −/− mouse embryonic fibroblasts are defective in chemotactic migration and cell spreading. Focal adhesions in these cells are larger than in normal cells suggesting a role in focal adhesion turnover, which is similar to that seen in FAK –/– cells and cells expressing dominant negative Src. Because FAK and Shp2 are shown to interact in normal fibroblasts, it is likely that Shp2 contributes to cell adhesion by regulating FAK and Src activity, possibly by dephosphorylating and activating Src. Not surprisingly, both Src and FAK expression are up-regulated in colon cancer and squamous cell carcimomas respectively.

Yet another phosphatase involved in the regulation of FAK activity and possibly other focal adhesion components is the tumour suppressor PTEN/MMAC1/TEP1 which is deleted or mutated in many advanced cancers. Aside from its role as a regulator of the PI 3-kinase pathway by dephosphorylation of 3′ phosphoinositides, PTEN is also a tyrosine phosphatase with FAK as its most notable substrate. Yamada (Bethesda) showed that in addition to regulation of integrin signalling to the MAP kinase pathway, PTEN determines the phosphorylation state of the adaptor, Shc, which is required for MAP kinase signalling from growth factor receptors. Interestingly, by tracking cell movements on fibronectin, Yamada was able to define two distinct but additive mechanisms of PTEN-regulated MAP kinase activation where Shc and MAP kinase are involved in random migration movements, whereas FAK and focal adhesion assembly are involved in directed cell movement. Perhaps the coordination of two such pathways is used by the cell to regulate the speed and direction of movement separately.

### CELL INVASION

The growth of benign tumours respects cellular boundaries, remaining confined to tissue compartments. During the transition to an invasive carcinoma, tumour cells actively penetrate the epithelial basement membrane and intermix with other cell types. Again the interplay between a cell and the extracellular environment will be crucial in facilitating cell invasion. The up-regulation and secretion of ECM-degrading proteases, in particular the matrix metalloproteinases (MMPs) are invariably linked to malignant progression. Stromelysin-1 (MMP-3) and gelatinase B (MMP-9) are expressed in the stromal compartment and are capable of degrading multiple ECM components. Transgenic mice carrying tetracycline-inducible expression of MMP-3 in the mammary gland have phenotypically normal epithelial glandular structures in the absence of MMP-3, but form invasive mesenchymal tumours when MMP-3 is expressed (Werb, San Francisco, CA). Importantly, once initiated, tumour formation becomes independent of MMP-3 expression indicating that MMP-3 functions in the early stages of tumour progression. Conversely, Matrilysin (MMP-7) is localized to the apical surface of epithelial cells and thus does not contact the basement membrane. Nevertheless MMP-7 overexpression in transgenic mice accelerates the formation of neu-induced metastatic tumours. Conversely, a reduction in tumours is seen in mice with a deleted APC gene when crossed with MMP-7 −/− mice (Matrisian, Nashville, TN). Significantly, these studies indicate that of the 20 or so MMP proteins, each appears to act in different cell types or at different stages of tumour progression.

The hyaluronan receptor, CD44, links the ECM to intracellular responses primarily through interaction with ERM (Ezrin-Radixin-Moesin) proteins which serve to link the plasma membrane to the intracellular cytoskeleton. Isacke (London) addressed the intracellular associations of CD44 with ezrin and the regulation of the interaction by phosphorylation events. Ezrin activation and localization to the membrane is dependent on unfolding of the protein through phosphorylation events. Ezrin is known to interact with a number of proteins including positive and negative regulators of Rho GTPase. Mangeat (Montpellier) mimicked this opened molecule by inserting Green Fluorescent Protein into the middle of Ezrin to induce its unfolding, as a means to further understand the functional determinants of the molecule. Herrlich (Karlsruhe) showed that another ERM protein, Merlin, also associates with CD44 and inhibits Schwannoma growth in nude mice. The extracellular domain of CD44 is proposed to regulate growth factor processing and presentation. CD44 interacts with regulatory components important for invasion including MMP-9 and growth factors, and is required for activation of the c-Met receptor and consequent scattering and invasion. CD44 plays a role in growth factor presentation by regulating the cleavage of the HGF precursor. The mechanisms of how CD44 does this is not known, however, CD44 is in close proximity with other molecules such as the urokinase-type plasminogen activator receptor which are required for growth factor activation. HGF-mediated cell scattering and invasion is blocked by CD44 antibodies; the same antibodies will also inhibit metastasis in mice.

It is clear, however, that cells will require the coordinated activity of many gene products in order to become invasive. This issue was addressed by Ozanne (Glasgow), who described the results of large scale screens designed to identify those genes which are required for or contribute to cell invasion as well as genes which need to be suppressed for transformation to occur. Based on the premise that the transcription factor, AP-1, controls expression of genes required for invasion, a number of AP-1-dependent gene products including CD44 and ezrin as well as many novel genes have been identified and verified as contributing to aspects of cell invasion.

### THE SIGNIFICANCE OF ANGIOGENESIS AND VASCULARIZATION IN CANCER PROGRESSION

Tumours require the formation of new blood vessels to support their growth (the process of angiogenesis), providing the basis for presentations by the keynote speaker Judah Folkman (Boston) and Rakesh Jain (Boston). Folkman emphasized the scope for therapeutic intervention provided by the many known natural activators (e.g. bFGF, VEGF) and inhibitors (e.g. Thrombospondin-1, Angiostatin) of angiogenesis. Significantly, the receptors for angiogenic factors remain to be identified and will undoubtedly provide additional levels of intervention once identified. Jain has developed a novel in vivo microscopy that allows direct

 © 2000 Cancer Research Campaign visualization of tumours and the measurement of events at the cellular level. This approach has provided powerful insight not only into angiogenesis and blood flow in tumours, but also leucocyte adhesion and vascular permeability. This methodology showed, for example, that the pore sizes in the walls of tumour blood vessels depend not only on tumour type, but also the site of tumour growth, factors which have important implications for the delivery of drugs and gene carriers to tumours.

Given that integrin alpha-v expression is up-regulated during angiogenesis and by angiogenic growth factors, and is the successful target of anti-angiogenic therapies, it was with some surprise that mice with targeted disruptions in all alpha-v integrins display normal angiogenesis and vascularization (Hynes, Cambridge, MA). While clarification of these discordant results awaits, it may be that other integrins such as $\alpha_5\beta_1$, $\alpha_1\beta_1$ and $\alpha_2\beta_1$ integrins, which also appear to be involved in angiogenesis, are able to compensate for the lack of alpha-v integrins in these mice.

### THERAPEUTIC STRATEGIES TO CIRCUMVENT METASTASIS

Successful metastatic progression requires the completion of all of the above described processes, providing many levels of attack for therapeutic intervention. In the long-term, those genes identified in the screens described by Ozanne will be of great informative value when arrayed onto microchips and used to screen tumours, thereby identifying new potential targets for intervention.

### Angiogenesis

The idea that successful metastatic progression is totally dependent on the formation of new blood vessels and a blood supply to support new tumour growth has vastly expanded the potential for new approaches to anticancer therapies. In addition, cancer therapy directed at non-malignant, and therefore genetically stable, endothelial cells reduces the chances of mutation to drug-resistant variants. Currently, there are 19 clinical trials underway in the USA aimed at various levels of angiogenesis, six of which are in phase III. Drugs designed to block endothelial cells include Endostatin, a C-terminal product of collagen XVIII, which inhibits endothelial cell proliferation and migration. Other modes of attack include blocking activators of angiogenesis such as the phase II trials underway using anti-VEGF antibodies in patients with metastatic kidney cancer.

### Drug design based on inhibitors of MMPs

A greater understanding of MMP function will undoubtedly point the way toward therapeutic intervention, targeted either at the proteinases themselves, or at new molecular targets downstream of the proteinases. A number of synthetic and naturally occurring MMP inhibitors are currently in clinical trials. One such endogenous inhibitor of MMP-3, TIMP-1 (tissue inhibitor of metalloproteinases-1), when overexpressed, is able to quench the ability of MMP-3 to promote neoplasia in transgenic mice. However, the work of Werb clearly shows that at least some MMPs, like MMP-3, function early in metastatic progression, and thus therapeutic targeting will be of little use once the tumour has progressed. In these cases, downstream targets of MMPs may be of greater clinical use.

While it is clear that the mechanisms of metastatic progression are highly complex and not completely understood, the optimism and enthusiasm generated by the progress presented at the Beatson Conference clearly points a way forward for future medical intervention.

© 2000 Cancer Research Campaign

